# Adenoid cystic Carcinoma and Carbon ion Only irradiation (ACCO): Study protocol for a prospective, open, randomized, two-armed, phase II study

**DOI:** 10.1186/s12885-021-08473-5

**Published:** 2021-07-15

**Authors:** Kristin Lang, Sebastian Adeberg, Semi Harrabi, Thomas Held, Meinhard Kieser, Jürgen Debus, Klaus Herfarth

**Affiliations:** 1 Department of Radiation Oncology, Heidelberg University Hospital, Heidelberg University, Im Neuenheimer Feld 400, 69120 Heidelberg, Germany; 2 Heidelberg Institute of Radiation Oncology (HIRO), Heidelberg, Germany; 3 Institute of Medical Biometry and Informatics, Heidelberg University, Heidelberg, Germany; 4 Heidelberg Ion-Beam Therapy Center (HIT), Department of Radiation Oncology, Heidelberg University Hospital, Heidelberg, Germany

**Keywords:** Adenoid cystic carcinoma, Carbon ions, Recurrence, Local control

## Abstract

**Background:**

Adenoid cystic carcinoma is a rare form of head and neck cancer with a slow, but aggressive growth pattern which remains a challenge for local tumor control. Based on phase II data, radiation treatment using partially high LET radiation results in a prolonged PFS and OS. There is a paucity of randomized clinical data examining the role of the use of high LET radiation only. Therefore, the purpose of this prospective clinical trial is to analyze local control rates in patients with node negative ACC treated with carbon ion radiotherapy alone compared to a combined modality approach.

**Methods:**

This trial is conducted as a prospective, open-label, phase II, two-armed, investigator-initiated study comparing the local control rates in node negative ACCs of the head and neck treated either with sole carbon ion radiotherapy or a combination of carbon ions and photons. Secondary outcomes investigated are progression-free survival, overall survival, acute and late toxicity, and quality of life. A total of 314 patients will be randomly assigned to C12 treatment alone or bimodal treatment: Patients in the experimental group will receive a dose of 51 Gy (RBE) in 17 fractions and a boost of 15 Gy (RBE) in 5 fractions. Patients in the control group will receive 25 fractions photon IMRT 50Gy and a boost using 8 × 3 Gy (RBE) carbon ions. Local control will be assessed in regular follow up examinations until 5 years after the completion of treatment.

**Discussion:**

The present trial aims to evaluate local control rates to compare sole carbon ion radiotherapy to bimodal radiotherapy with carbon ions and photons in patients with node negative ACCs of the head and neck region. Local control is selected as the primary endpoint due to its major clinical relevance because of slow but aggressive growth patterns.

**Trial registration:**

The study was prospectively registered on 2nd January 2020: ClinicalTrials.gov, NCT04214366. “Adenoid Cystic Carcinoma and Carbon Ion Only Irradiation (ACCO)”.

**Study status:**

Under recruitment, participant recruitment is not completed. Start of recruitment was January 2020. There are no results been published or submitted to any journal.

**Supplementary Information:**

The online version contains supplementary material available at 10.1186/s12885-021-08473-5.

## Background

Adenoid cystic carcinomas (ACCs) are rare tumors, which occur mostly in the head and neck region and account for about 10–15% of malignant salivary gland tumors. They grow slowly; however, due to their growth pattern exhibit a tendency for local recurrences. For this reason, postoperative irradiation is indicated, especially in the presence of risk factors (locally advanced (pT3/4), positive postoperative microscopic or macroscopic margins (R1/2) or perineural spread (Pn1)). Lymphatic metastases are detected in 19% of salivary gland ACCs [1] and in only 5% in ACCs located in the nasopharynx, paranasal sinuses, lacrimal glands, or external auditory canal [2]. Most common hematogenous metastasis occurs in the lungs with an incidence of 35–50% [3]. Other sites of metastasis are liver and bone. Due to the rarity of ACC, reports of these tumors are usually retrospective analyses by individual clinics or institutes, extending over a very long period of time with changing radiation technology.

Using modern photon techniques with a median dose of 66 Gy in 1.8–2.0 Gy/fraction resulted in local control rates of 59 and 40% at 3 years and 5 years, respectively, at a median follow-up time of 63 months. Almost one third of patients had acute grade 3 mucosal toxicity, with 3% reporting higher-grade late radiation effects [4].

Other radiation techniques using neutrons, known to have a high linear energy transfer (LET) were considered for a long time as the primary treatment option for patients with ACCs because of historic local control rates of around 75%. Yet, the main problem with neutron irradiation is the excessive late grade 3/4 toxicities in about 20% patients [5].

Initial data using combined treatment strategies with intensity-modulated radiotherapy (IMRT) and a 6–8 fraction carbon ion (C12) boost of 3 Gy (RBE) single doses was published in 2005 and showed improved local control rates compared to historical IMRT techniques alone [6]. Updated data 10 years later showed progression free survival rates of 84% at 3 years and 60% after 5 years, at a median follow-up time of 74 months. This also reflected a significant survival benefit: median overall survival in the combination treatment was 102 months versus 74 months after IMRT alone. Severe grade 3 toxicities included mucositis in 25% and dermatitis in 10%. Late complications included unilateral hearing loss in 10%, anosmia in 10% and a unilateral blindness due to retinal detachment in one patient [4, 7, 8]. Given these results, bimodal therapy with carbon ions is now regarded as standard of care in centers, where carbon ion irradiation is available.

Data examining the role of carbon ions alone in the treatment of ACC has been collected in Japan, but there are no randomized data available. Treatment in Japan was delivered with passive beam modulation. To calculate the biological dose, dose profiles were performed assuming a fixed spread out Bragg peak (SOBP). The biological dose calculation of Japanese treatments at the Heavy-Ion Medical Accelerator (HIMAC) differs from calculation in Europe where mostly the local effect model (LEM) for carbon irradiation with active scanning is used [9]. In addition, irradiation in Japan was carried out with 3–4 fractions per week, whereas treatment in Europe is most commonly delivered 5–6 days/week.

Mizoe et al. published data on dose escalation in a group of patients with salivary gland tumors, of which 68% were ACC histology [10]. Dose was increased from 18 × 2.7 Gy (RBE HIMAC) to 18 × 3.9 Gy (RBE HIMAC) with 3 exposures per week. In a later group, fractionating was increased to 16 fractions (4x / week) with single dose between 3.3–4.0 Gy. The local control rates of these two groups did not differ significantly, reaching 75% at 3 years, 65% at 4 years and 60% at 5 years. Acute grade 2 or higher mucosal toxicities were described in only one patient. No patient developed higher grade late toxicities. In a later series of 113 patients with ACC, 16 fractions of 3.7–4.0 Gy(RBE HIMAC) were administered; here, 3-, 4-, and 5-year local control rates of 89, 82 and 69% were observed. One third of patients (37 in total) exhibited acute grade 3 toxicities of the mucous membranes. Late radiation effects ≥ grade 3 were reported in 24% of the patients [11].

## Methods/design

### Trial aim

The purpose of this trial is to randomly asses the loco-regional control rates in patients with node negative ACCs of the head and neck region treated with sole radiotherapy of carbon ions compared to bimodal radiotherapy.

We propose that patients treated with carbon ions alone have better local control rates compared with patients treated with standard bimodal radiotherapy.

### Trial design

The ACCO trial is a single center, prospective, randomized, two-arm phase II study. The trial has been designed by the study initiators at the Department of Radiation Oncology of the University of Heidelberg. The trial is carried out at the University of Heidelberg, Department of Radiation Oncology. The University Heidelberg is responsible for trial management and coordination, as well as quality assurance including reporting, monitoring and database management. The current version of study protocol is version 1.4 from March, 06th 2019 (supplementary material 1).

**Fig. 1:**
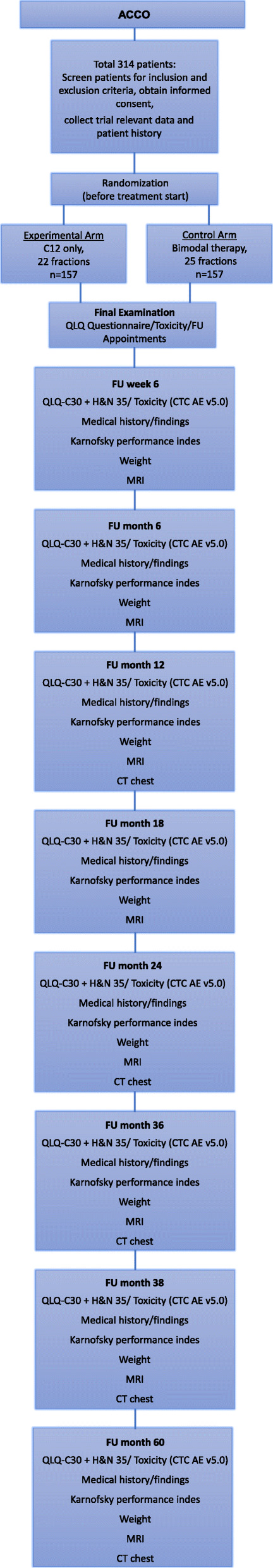
Study workflow and treatment arms

The study workflow and treatment arms are depicted in Fig. 1.

Three hundred fourteen patients with ACC of the head and neck region fulfilling the inclusion criteria will be enrolled in this phase II clinical trial.

### Inclusion criteria

Patients meeting the following criteria will be included in the trial:

- Age 18–80 years
- Karnofsky Performance Score > 60% or ECOG 0/1 (minimum: self-sufficiency, normal activity or work not possible)
- Histologically confirmed adenoid cystic carcinoma in the head and neck area
- Indication for irradiation:
  - non-operable and/or.
  - R1/R2 resected and/or.
  - perineural sheath invasion (PNI+) and/or.
  - pT3/pT4.
  - written informed consent.
  - Adequate contraception.

### Exclusion criteria

Patients presenting with one of the following criteria will not be included in the trial:

- Rejection of the study by the patient
- Patient is not able to consent
- Stage IV (distant metastases), with exception of pulmonary metastases of ≤1 cm
- Lymph node involvement (clinical or pathological)
- Previous radiotherapy in the head and neck area
- Active medical implants for which there is no ion radiation authorization at time of treatment (e.g., cardiac pacemaker, defibrillator)
- Contraindication to MR imaging
- Simultaneous participation in another clinical study that could influence the outcome of this study or the other study

### Randomization

After meeting eligibility criteria, 314 patients will be randomly assigned to C12 treatment alone or combination treatment. To obtain comparable treatment groups with respect to known and unknown risk factors, each patient becomes randomized to the treatment groups in balanced permutated blocks and stratified for residual tumor and lung metastases using the web-based software randomizer operated by the Institute of Medical Informatics, Statistics and Documentation of the Medical University of Graz (https://www.randomizer.at).

### Study treatment

#### Experimental group

Sole C12 Radiotherapy with a target dose of 51 Gy (RBE) in the basic plan and an additional dose of 15 Gy (RBE) to the boost volume (Table 1).

**Table 1:** Target doses referred to 95% of the volume. For carbon ions, the biological dose is based on the LEM1 model using an alpha/beta of 2Gy. The biologically effective dose in 2 Gy fractions (BED2Gy) is calculated assuming an alpha/beta value of 3 Gy for ACC tissue

	Experimental arm C12-only RT		Control arm Bimodal RT (photon IMRT & C12)	
	CTV_basic	CTV_boost	CTV_basic	CTV_boost
**Single dose**	3Gy(RBE)	3Gy(RBE)	2Gy	3Gy(RBE)
**Total dose**	51Gy(RBE)	15Gy(RBE)	50Gy	24Gy(RBE)
**BED2Gy**	61Gy	18Gy	50Gy	29Gy

#### Conventional group

Bimodal treatment with a target dose of 50Gy photons in the basic plan and an additional dose of 24 Gy (RBE) C12 to the boost volume (Table 1).

### Trial objectives

The primary objective is to demonstrate increase loco-regional control rates in patients with node negative ACCs treated with radiotherapy of carbon ions only compared to bimodal radiotherapy in this tumor entity after 5 years.

Secondary objectives are progression-free survival (time from randomization until local or distant tumor progression/occurrence of distant metastases, death without prior local progression, or end of follow-up) after 3 and 5 years, OS (time from randomization until death or end of follow-up) after 3 and 5 years, acute and chronic toxicity, quality of life (QoL). Toxicity and QoL assessment are performed according to international validated scores and questionnaires (QLQ-C30 and QLQ-H&N35, CTC AE 5.0) (Table 2).

**Table 2:** Follow-up Workflow

	IE	FE	FU week 6	FU month 6	FU month 12	FU month 18	FU month 24	FU month 36	FU month 48	FU month 60
**In−/exclusion criteria fulfilled**	x									
**Informed consent**	x									
**Medical history/findings**	x		x	x	x	x	x	x	x	x
**Karnofsky performance index**	x		x	x	x	x	x	x	x	x
**Height**	x									
**Weight**	x		x	x	x	x	x	x	x	x
**QLQ-C30 and QLQ-H&N35**	x	x	x	x	x	x	x	x	x	x
**Symptoms/Toxicities** **(CTC AE v5.0)**	x	x	x	x	x	x	x	x	x	x
**Documentation staging examination**	x									
**MRI**	x		x	x	x	x	x	x	x	x
**CT chest**	x				x		x	x	x	x
**CT/Sonography Liver**	x									
**Bone scintigraphy**	x									
**Scheduling FU**		x								

*IE* Initial examination; *FE* final examination of RT; *FU* follow up

### Treatment planning and radiation therapy

#### Experimental and conventional arm

Radiotherapy is administered after full recovery from surgical resection or in prior inoperable situations. Patients are immobilized using a thermoplastic mask. Computed tomography (CT) must be performed without contrast enhancement using a slice thickness of 3 mm and if possible also with contrast medium. Dose constraints of normal tissue will be respected to QUANTEC reports [12, 13]. The maximum exposure of the organs at risk should not exceed the TD 5/5 (toxic dose that causes 5% serious complications in 5 years) of the respective organs. The protection of the spinal cord, chiasm and brain stem is a high priority. The preservation of the respective optic nerves should be accomplished according to the initial tumor spread in consultation with the patient. Dose constraints of normal tissue will be respected according to Table 3.

**Table 3:** Dose constraints of normal tissue. The biologically effective dose in 2 Gy fractions (BED2Gy) is calculated assuming an alpha/beta value of 2Gy for all normal tissues. *ALARA* As low as reasonable achievable. Dose constraints of can be exceeded in case of tumor involvement and with agreement of the patient

Organ at risk	Dose contraints	BED2Gy	
**Brain stem**	54Gy(RBE)/82%	60Gy	Dmax superficial
**Chiasma**	49.5Gy(RBE)/75%	52.6Gy	Dmax
**Optic nerves**	49.5Gy(RBE)/75%	52.6Gy	Dmax
**Spinal cord**	45Gy(RBE)/68%	45.3Gy	Dmax
**Parotid gland**	31Gy(RBE)/47%	26.4Gy	Dmean
**Lower jaw**	60Gy(RBE)/90%	69.8Gy	Dmax superficial
**Lens**	ALARA		
**Bulb**	ALARA		
**Lacrimal gland**	ALARA		
**Inner ear**	ALARA		
**Temporomandibular joint**	ALARA		

### Experimental arm

Radiation therapy is performed using carbon ions alone with 17 fractions and a target dose of 51 Gy (RBE) in basic plan and 5 fractions with an additional target dose of 15 Gy (RBE) to the boost volume (Table 1). The dosages refer to 95% of the volume. For carbon ions, the biological dose is based on the LEM1 model, taking into account an alpha/beta value of 2Gy for all tissues.

### Target definition

GTV: Macroscopic tumor extension according to MRI at the time of treatment planning.

GTV initial: preoperative tumor in operated patients.

CTV_boost: GTV and possibly GTV initial plus 6 mm margin (9 mm along the perineural spreading path), considering anatomical boundaries.

CTV_basic: CTV_boost plus 6 mm margin (12 mm along the perineural spreading path), considering anatomical boundaries; thereby partially included lymph node stations should be included completely.

### Conventional arm

Radiation therapy is performed using bimodal treatment with 25 fractions photons and a target dose of 50Gy in basic plan and additional 8 fractions carbon ions with a target dose of 24 Gy (RBE) to the boost volume (Table 1). The dosages refer to 95% of the volume.

For carbon ions, the biological dose is calculated using the LEM1 model, taking into account an alpha/beta value of 2Gy for all tissues.

### Target definition

GTV: Macroscopic tumor extension according to MRI at the time of treatment planning.

GTV initial: preoperative tumor in operated patients.

CTV_boost: GTV and possibly GTV initial plus 6 mm margin (9 mm along the perineural spreading path), considering anatomical boundaries.

CTV_basic: CTV_boost plus 3 mm margin and draining lymph nodes (level II and III only), in case of crossing the mid line, bilateral.

### Follow up

The baseline visit will be performed after enrolment of the patient into the trial. During the initial examination (IE) a clinical assessment, staging examination (CT chest, MRI, sonography/CT liver, bone scintigraphy) as well as analysis of QoL is scheduled. Both study groups will be evaluated with final examination (FE) at the last treatment day to assess the potential toxicities and QoL (secondary endpoint). The second, third, fourth, fifth, sixth, seventh, eighth and ninth study visits are planned 6 weeks, 6 months, 12 months, 18 months, 24 months, 36 months, 48 months and 60 months (primary and secondary endpoints) after the treatment start. These visits will include a clinical assessment, analysis of QoL (QLQ-C30 and QLQ-H&N35) as well as MRI scans. CT chest scans will be performed once a year. The follow-up workflow is depicted in Table 2.

### Outcome measures

#### Primary endpoint

The primary hypothesis of the trial is a greater loco-regional control rate 5 years after radiotherapy with C12 ions only. Locoregional tumor control will be assessed with MRI imaging up to 5 years after treatment start.

#### Secondary endpoints

Secondary analysis includes regional and distant recurrence rates, disease-free as well as overall survival, assessment of acute and chronic toxicity (CTCAE v5.0) and QoL (QLQ-C30 and QLQ-H&N35).

### Statistical analysis and methods

The primary alternative hypothesis of the trial is that there is a difference between the two treatment arms with respect to the primary endpoint, defined as a greater local control rate 5 years after radiotherapy with sole C12 ions. Control rates of approximately 60% were observed with treatment with IMRT and C12 Boost, and it is estimated that this rate can be improved by 10% using sole C12 [4, 7, 8]. A logistic regression model which is adjusted for the factors residual umor and lung metastases present is applied in the analysis. The null-hypothesis is tested at the one-sided level α = 0.15. Under these assumptions, a number of 266 patients (133 per arm) is necessary to achieve a power of 75% with the Chi^2^ test; it can be assumed that the power for the logistic regression model used in the analysis at least as high as this value.

Assuming a drop-out rate of 15%, 314 patients will be included in the study. The calculations were done with nQuery Advisor version 7.0. The increased significance level compared to a confirmatory Phase II study on the one hand considers the phase II character of the study and, on the other hand, the number of patients that can be recruited in a manageable time leading to an acceptable power. In addition to the results of statistical testing, the odds ratio for the treatment effect with respect to the primary endpoint are provided together with the two-sided 70% confidence interval. The time-to-event curve for the main objective criterion is estimated using the Kaplan and Meier method [14]. The primary analysis is performed based on the full analysis set which includes all randomized patients that will be analyzed according to the intention-to-treat (ITT) principle. In addition, a per-protocol population (PP) analysis is performed as a sensitivity analysis. The secondary objective criteria are evaluated by methods of descriptive data analysis [15]. Corresponding to the scaling level, suitable measures of the empirical distribution are computed for ordinal and interval-scaled variables. For nominally scaled endpoints, absolute and relative frequencies are calculated. For time-to-event endpoints, the Kaplan-Meier method is used to estimate the probability of each occurring target event as a function of time. In addition, two-sided *p*-values and 95% confidence intervals – for time-to-event endpoints with adequate consideration of censored observations – will be calculated and reported which are to be interpreted as descriptive measures. Statistical analysis is based on the International Conference on Harmonization Guidelines “Structure and Content of Clinical Study Reports” and “Statistical Principles for Clinical Trials”. Analyses will be conducted using SAS v9.4 (SAS Institute, Cary, NC).

### Ethical issues, information, and safety

The study protocol, Patient Information sheet, and Declaration of Informed Consent and consent to participate was approved by the Heidelberg University Ethics Committee (S-010/2018).

The Ethics Committee will be promptly informed by the principal investigator of any changes in the study protocol that may affect patient safety. The procedures described in the submitted study protocol regarding the performance, evaluation, and documentation of this study has been selected in such way that the principles of the Good Clinical Practice (GCP) guidelines are observed. The regulations regarding medical confidentiality will be obtained from all participants in the study. This study complies with the Helsinki Declaration. The statutory requirements of the Radiation Protection Ordinance (StrSchV), the X-ray Ordinance (RöV) and the Directive on Radiation Protection (Richtlinie zum Strahlenschutz) are complied with. The principal investigator has at least 2 years of experience in clinical trials. The study was reported to the Federal Office for Radiation Protection (Bundesamt für Strahlenschutz (BfS). A radiation protection insurance is issued for the patients.

### Availability of data and materials

The data is collected, managed and processed electronically in the in-house research database. To ensure data quality and consistency, internal quality control measures will be carried out.

The originals of all study documents are kept at the Study Center for at least 30 years after the final report has been prepared.

The dataset used and analyzed during the current study will be available from the corresponding author upon reasonable request.

Regulatory authorities may request access to all source documents, CRF and other trial documentation.

### Trial status

Recruiting.

## Discussion

The primary aim of this trial is to investigate the effect of carbon ion irradiation alone compared to standard irradiation using a combination of photon IMRT and carbon ion treatment in patients with node negative ACCs of the head and neck area.

Initial data using combined treatment strategies with photons and carbon ion boost were published in 2005 by Schulz-Ertner et al., which showed improved local control rates compared to historical IMRT techniques with photons alone [6, 16]. Photon therapy alone resulted in minor local control rates at 5 years between 38 and 64% [17, 18]. Compared with modern photon RT, the LC rates in combined-modality approach were 77.5% versus 24.9% at 2 years. However, because of the small patient numbers and short follow-up, the difference was not statistically significant at the time [6]. In a pilot project updated data for inoperable and subtotally resected patients with ACCs of the head and neck region between 1998 to 2008 showed progression free survival rates of 60% for the combined treatment group after 5 years and led to the establishment of this regimen as the treatment of choice in Germany [7, 16, 19].

Other irradiation techniques using neutron ion beams with a high linear energy transfer (high-LET) were considered for a long time as primary treatment option for patients with ACCs given local control rates of up to 75%, but the main problems of neutron irradiation were late toxicities grade 3/4 in about 20% patients [5].

Currently, the randomized data for sole treatment with carbon ions of ACCs are lacking while there are Japanese single arm studies available [10, 11]. Heidelberg University Hospital has gained experience with sole carbon ion irradiation in the setting of tumor recurrence after previous irradiation with a local control rate of 70% after 1 year [7].

The primary goal of the ACCO study is to show that carbon ion irradiation alone results in a significantly better local control rate compared to standard combination treatment in patients with ACCs of the head and neck area.

## Supplementary Information

**Additional file 1.**

## Data Availability

All data generated or analyzed during this study are included in this published article.

## Abbreviations

ACC: Adenoid cystic carcinoma
ACCO: Adenoid cystic carcinoma and carbon ion only irradiation
BED: Biologically effective dose
BfS: Bundesamt für strahlenschutz (federal office for radiation protection)
C12: Carbon ions
CT: Computed tomography
CTV: Clinical target volume
DSMB: Data and safety monitoring board
ECOG: Eastern cooperative oncology group
FE: Final examination
FU: Follow up
Fx: Fractionation
GSI: Gesellschaft für schwerionenforschung
GTV: Gross tumor volume
HIMAC: Heavy-ion medical accelerator
HIT: Heidelberg ion beam therapy center
IE: Initial examination
IMRT: Intensity-modulated radiotherapy
LC: Local control
LEM: Local effect model
LET: Linear energy transfer
MRI: Magnetic resonance imaging
QLQ-C30: Quality of life questionnaires - general
QLQ-H&N35: Quality of life questionnaires - head and neck
RBE: Relative biological effectiveness
SOBP: Spreadout bragg peak
TD: Total dose
tox: Toxicity
