# Supplementary material for: Adenoid cystic Carcinoma and Carbon ion Only irradiation (ACCO): Study protocol for a prospective, open, randomized, two-armed, phase II study
Source: BMC Cancer. 2021 Jul 15;21:812. doi: 10.1186/s12885-021-08473-5 (PMC8281682; doi:10.1186/s12885-021-08473-5)
Supplement: Supplementary file 1 — Additional file 1. [file 12885_2021_8473_MOESM1_ESM.pdf]

Studienprotokoll

Adenoid cystic Carcinoma and Carbon ion Only irradiation

ACCO-Studie

- prospektive, offene, randomisierte, zweiarmige, Phase II Studie -

Version 1.4.2 vom 30.01.2020

Studienleiter
Prof. Dr. Klaus Herfarth
Universitätsklinikum Heidelberg
Klinik für RadioOnkologie und Strahlentherapie (Czerny-Klinik)
Im Neuenheimer Feld 400
69120 Heidelberg

Der Inhalt von Protokoll und Prüfbogen sind vertraulich zu behandeln und dürfen ohne Zustimmung der Studienleitung weder mündlich noch schriftlich an Unbeteiligte weitergegeben werden.

Durchführende Einrichtung:
Klinik für Radioonkologie und Strahlentherapie
Ärztlicher Direktor: Prof. Dr. Dr. Jürgen Debus
Universität Heidelberg
Im Neuenheimer Feld 400, 69120 Heidelberg
Tel.: +49-6221-56 8201
Fax: +49-6221-56 5353
juergen.debus@med.uni-heidelberg.de

Studienleitung:
Prof. Dr. Klaus Herfarth
Stellvertretender Ärztlicher Direktor
Klinik für Radioonkologie und Strahlentherapie
Universität Heidelberg
Im Neuenheimer Feld 400, 69120 Heidelberg
Tel.: +49-6221-56 8202
Fax: +49-6221-56 5353
klaus.herfarth@med.uni-heidelberg.de

Studienkoordination:
Dr. Sebastian Adeberg
Klinik für Radioonkologie und Strahlentherapie
Universität Heidelberg
Im Neuenheimer Feld 400, 69120 Heidelberg
Tel.: +49-6221-56 8202
Fax: +49-6221-56 5353
sebastian.adeberg@med.uni-heidelberg.de

Biometrie:
Prof. Dr. Meinhard Kieser
Geschäftsführender Direktor
Institut für Medizinische Biometrie und Informatik
Universität Heidelberg
Im Neuenheimer Feld 130.3, 69120 Heidelberg
Tel.: +49-6221-56 4140
Fax: +49-6221-56 4195
meinhard.kieser@imbi.uni-heidelberg.de

Studiensekretariat:
Karen Lossner
Klinik für Radioonkologie und Strahlentherapie
Universität Heidelberg
Im Neuenheimer Feld 400, 69120 Heidelberg
Tel.: +49-6221-56 37748
Fax: +46-6221-56 1979
karen.lossner@med.uni-heidelberg.de

Data safety monitoring board:

Prof. Dr. Marc Münter
Katharinenhospital
Kriegsbergstr. 60
70174 Stuttgart
Tel.: +49-711-278 34201
Fax: +49-711-278 34209
m.muenter@klinikum-stuttgart.de

Prof. Dr. Florian Sterzing
Strahlentherapie Süd
Am Klinikum Kempten
Robert-Weixler-Straße 50
87439 Kempten
Tel.: +49-831-960 7940
Fax: +49-831-960 7943456
f.sterzing@strahlentherapie-kempten.de

Inhaltsverzeichnis

1.0 Synposis 6

2.0 Zusammenfassung 11

3.0 Hintergrund 11
3.1 Radiotherapie mit Photonen 12
3.2 Radiotherapie mit Neutronen 12
3.3 Radiotherapie mit kombinierter Modalität (IMRT + C12) 12
3.4 Kohlenstoffionentherapie 13

4.0 Studienziele 14
4.1 Primäres Studienziel 14
4.2 Sekundäre Studienziele 14

5.0 Studiendesign 14

6.0 Teilnehmende Prüfärzte/Prüfzentren 15

7.0 Auswahl der Patienten 16
7.1 Einschlusskriterien 16
7.2 Ausschlusskriterien 16

8.0 Patientenaufnahme/Registrierung 17

9.0 Behandlungsplan 18
9.1 Strahlentherapie 18
9.2 Bestrahlungsplanung 18
9.3 Konturierung 18
9.4 Dosisspezifikation 19
9.5 Risikoorgane 20
9.6 Kontrollen am Bestrahlungsgerät 21
9.7 Supportivmaßnahmen während der Strahlentherapie 21

10.0 Klinische Untersuchungen (Visiten) 22
10.1 Übersicht 22
10.2 Eingangsuntersuchung 22
10.3 Abschlussuntersuchung 22
10.4 Follow-Up 23

11.0 Dauer der Studienteilnahme 24
11.1 Vorzeitiges Ausscheiden eines Patienten aus der Studie 24

12.0 Erfassung der therapeutischen Effektivität 24
12.1 Zeitplan 24
12.2 Definition eines Rezidives 24

13.0 Sicherheit 25
13.1 Ermittlung der Sicherheit 25
13.2 Data Safety Monitoring Board 25

14.0 Dauer der Studie/Studienabbruch 26
14.1 Reguläres Studienende 26
14.2 Vorzeitiges Studienende/Abbruch der gesamten Studie 26
14.3 Individuelle Abbruchkriterien 26

15.0 Biometrie 27

15.1 Fallzahlberechnung 27
15.2 Randomisierung 27
15.3 Studienpopulation 27
15.4 Auswertung 28

16.0 Datenmanagement 29
16.1 Patientenidentifikationsliste 29
16.2 Datenerhebung/Dokumentationsbögen 29
16.3 Aufbewahrung der Studienunterlagen 29
16.4 Datenschutz 29

17.0 Ethische Grundlagen 31
17.1 Deklaration von Helsinki 31
17.2 Ethikkommission 31
17.3 Aufklärung der Patienten 31
17.4 Strahlentherapeutische Aufklärung 31
17.5 Verwendung, Speicherung und Weitergabe von Daten 31

18.0 Gesetzliche und administrative Regelungen 32
18.1. GCP 32
18.2 Gesetzliche Grundlagen 32
18.3 Strahlenschutz und Versicherung 32
18.4 Finanzierung 32
18.5 Publikation 32
18.6 Einhaltung des Protokolls und Protokolländerungen 32

19.0 Literatur 33

20.0 Anhang 35

1.0 Synposis

ACCO-Studie Synopsis	
Titel	**A**denoid cystic **C**arcinoma and **C**arbon ion **O**nly irradiation
Akronym	ACCO
Studienleitung	Prof. Dr. Klaus Herfarth<br>Klinik für Radioonkologie und Strahlentherapie<br>Universitätsklinikum Heidelberg
Studienkoordination	Dr. Sebastian Adeberg<br>Klinik für Radioonkologie und Strahlentherapie<br>Universitätsklinikum Heidelberg
Biometrie	Prof. Dr. Meinhard Kieser<br>Institut für Medizinische Biometrie und Informatik<br>Universitätsklinikum Heidelberg
Studienphase/-typ	Prospektive offene Phase II / Investigator-Initiated Studie
Geplanter Studienbeginn	Q2 2019
Studienende	Einschluss des letzten Patienten: Q2 2026
	Ende der Nachbeobachtungszeit: Q2 2031
Studienziel	Primäres Studienziel:<br>Erfassung der lokoregionäre Kontrollrate nach 5 Jahren
Studiendesign	Monozentrische prospektive offene, randomisierte, zweiarmige Phase-II-Studie
Geplante Patientenzahl	314 Patienten
Einschlusskriterien	• Histologisch gesichertes adenoidcystisches Karzinom im Kopf-Hals Bereich<br>• Indikation zur Bestrahlung:<br>nicht-operabel *und/oder*<br>R1/R2 reseziert *und/oder*<br>Perineuralscheiden Invasion (Pn+) *und/oder*<br>pT3/pT4<br>• Patientenaufklärung und schriftliche Einwilligung<br>• KI >60% oder ECOG 0/1 (mindestens: Selbstversorgung. Normale Aktivität oder Arbeit nicht möglich)<br>• Alter 18-80 Jahre<br>• Ausreichend wirksame Empfängnisverhütung

ACCO-Studie Synopsis	
Ausschlusskriterien	• Ablehnung der Studie durch den Patienten<br>• Patient ist nicht einwilligungsfähig<br>• Stadium IV (Fernmetastasen) mit Ausnahme von pulmonalen Metastasen ≤ 1 cm<br>• Lymphknotenbefall (klinisch oder pathologisch)<br>• Frühere Strahlentherapie im Kopf-Hals Bereich<br>• Aktive Medizin-Implantate, für die zum Zeitpunkt der Behandlung keine Genehmigung für eine Ionenbestrahlung existiert (z.B. Herzschrittmacher, Defibrillator,...)<br>• Kontraindikation gegenüber MRT Bildgebung<br>• Gleichzeitige Teilnahme an einer anderen klinischen Studie, die die Ergebnisse dieser Studie oder der anderen Studie beeinflussen könnte
Beschreibung der Therapie	Lokoregionäre Strahlentherapie mit Kohlenstoffionen (C12) (experimenteller Arm A) versus Photonen-IMRT mit Kohlenstoffionen-Boost (Kontrollarm B).
Definition des Zielvolumens	Primärtumorregion mit klinischem Sicherheitssaum unter Berücksichtigung der perineuralen Ausbreitungswege
Risikoorgane	Hirnstamm, Rückenmark, Innenohr, Temporalpole, Chiasma opticum, Sehnerven bds., Augenbulbus, Linse, Tränendrüse, Kiefergelenke, Gll. Parotis, Gll. submandibularis
Geplante Behandlungsdauer pro Patient	Bestrahlungsplanung: 2 Wochen<br>Bestrahlungsdauer: ~4 Wochen (22 Fraktionen) experimenteller Arm A<br>~6 Wochen (33 Fraktionen) Kontrollarm (Arm B)<br>Nachbeobachtung: 60 Monate ab Bestrahlungsbeginn
Durchführung der Therapie	Ionenstrahl-Therapiezentrum des Universitätsklinikums Heidelberg in Marburg oder Heidelberg (MIT oder HIT)<br>Die Behandlung wird ambulant durchgeführt.

ACCO-Studie Synopsis	
Checkliste vor Beginn der Therapie	• Schriftliches Einverständnis für die Teilnahme an der Studie<br>• Aktuelle MRT Bildgebung inkl. T1/T2 Fat-Sat Aufnahmen (nicht älter als 2 Monate)<br>• CT Thorax (nicht älter als 3 Monate)<br>• CT/Sonographie Leber (nicht älter als 3 Monate)<br>• Knochenszintigraphie (optional)<br>• Anamnese und körperlicher Befund (Gewicht, Größe, Karnofsky-Index)<br>• Lebensqualität QLQ-C30 und QLQ-H&N35 Fragebögen<br>• Zahnsanierung und Anfertigung einer Fluoridierungsschiene bei Bestrahlungen im Kieferbereich<br>• Randomisierung
Untersuchungen während der Bestrahlung	Dokumentation von klinischen Symptomen und Toxizität (nach den NCI CTCAE Version 5.0 Kriterien)
Nachuntersuchungen	6 Wochen nach Bestrahlungsende, danach 6 Monate nach Bestrahlungsbeginn und weiter halbjährlich in den ersten zwei Jahren, jährlich im 3.-5. Jahr<br>Nachsorgen inklusive klinischer Status, MRT, Erfassung der Toxizität und Lebensqualität, CT Thorax (1x jährlich ab Follow-up Monat 12)
Hauptzielkriterium	Lokale Kontrollrate nach 5 Jahren
Nebenzielkriterien	Progressionsfreies Überleben (5 Jahre)<br>Gesamtüberleben (5 Jahre)<br>Akuttoxizität (CTC AE V5.0)<br>Spättoxizität (CTC AE V5.0)<br>Lebensqualität (QLQ-C30 und QLQ-H&N35)

ACCO-Studie Synopsis	
Studienpopulationen	Die ITT-Population umfasst alle randomisierten Patienten, welche in jenem Therapiearm ausgewertet werden, zu dem sie randomisiert worden sind. Die ITT-Population ist das primäre Auswertungskollektiv für alle Wirksamkeitsendpunkte und Patientencharakteristika.<br>Per-Protokoll-Population (PP): Alle Patienten der ITT-Population, welche die geplante Therapie vollständig erhalten haben und deren Dokumentation zumindest soweit vollständig ist, dass aus den Daten Schlussfolgerungen über die Wirksamkeit abgeleitet werden können. Die Analysen der PP-Populationen dienen als Sensitivitätsanalysen, anhand deren die Robustheit der Ergebnisse aus der ITT-Population untersucht werden soll.<br>Safety-Population: Alle Patienten der ITT-Population, bei denen die geplante Therapie begonnen wurde (mind. 1 Tag). Patienten in der Safety-Population werden in jenem Therapiearm ausgewertet, gemäß dem sie behandelt worden sind. Dies ist das primäre Auswertungskollektiv für sämtliche Safety-Endpunkte.

ACCO-Studie Synopsis	
Biometrische Methoden	Primäres Studienziel ist der Nachweis, dass bei lokoregionärer Radiotherapie mit C12 Ionen die lokale Kontrollrate nach 5 Jahren π_{C12} größer als die lokale Kontrollrate der bimodalen Radiotherapie π_{bim} ist. Die Nullhypothese (H_0: $\pi_{C12} \leq \pi_{bim}$; „die lokale Kontrollrate nach 5 Jahren bei lokoregionärer Radiotherapie mit C12 Ionen ist kleiner oder gleich der lokalen Kontrollrate bei bimodaler Radiotherapie“) wird zum einseitigen Niveau α=0,15 getestet, für die Alternative einer lokalen Kontrollrate nach 5 Jahren bei lokoregionärer Radiotherapie mit C12 Ionen von π_{C12} =70% und bei bimodalen Radiotherapie von $\pi_{bim} = 60\%$ soll eine Power von 0,75 bestehen. Hierzu ist unter der Verwendung eines Chi²-Tests eine Fallzahl von 266 Patienten (133 pro Arm) notwendig. Unter der Annahme einer Drop-out-Rate von 15% werden 314 Patienten in die Studie eingeschlossen.<br><br>Der primärer Endpunkt „Lokoregionäre Tumorkontrolle gemäß MRT Bildgebung nach 5 Jahren“ wird mittels einem logistischen Regressionsmodell ausgewertet, welches für die Faktoren („Resttumor vorhanden“ (ja/nein) und „Lungenmetastasen vorhanden“ (ja/ nein) adjustiert. Die Nullhypothese wird hierbei zum einseitigen Niveau von α=0,15 getestet. Zusätzlich wird das zugehörige Odds Ratio für den Therapieeffekt mit einem zweiseitigen 70%-Konfidenzintervall angegeben. Fehlende Werte des primären Endpunkts werden mittels multipler Imputation ersetzt. Die Nebenzielkriterien werden mit Methoden der deskriptiven Datenanalyse ausgewertet. Ergänzend zur Angabe geeigneter zusammenfassender Maßzahlen der empirischen Verteilung werden deskriptiv zu interpretierende zweiseitige 95%-Konfidenzintervalle angegeben.
Studienende	Die Studie endet mit der Follow-up Visite in Monat 60 nach Einschluss des letzten Patienten.

2.0 Zusammenfassung

Adenoidzystische Tumore (ACC) sind seltene Tumore der Kopf-Hals Region, die ein langsames Wachstum aufweisen. Das perineurale Wachstumsmuster führt bei lokal fortgeschrittenen Tumoren nach einer Operation zu einer hohen Rezidivrate, so dass diese Tumoren häufig postoperativ bestrahlt werden müssen, wenn sie nicht bei Inoperabilität schon einer primären Strahlentherapie zugeführt werden. ACCs gehören zu den strahlenresistenten Tumoren und bedürfen einer hohen Strahlendosis. Die bisher besten Tumorkontrollen wurden mittels Neutronenbestrahlung erzielt. Neutronen gehören zu den sog. Hoch-LET (linearer Energietransfer) Strahlen, die Strahlenresistenzen überwinden können. Nachteil der Neutronenbestrahlung war eine hohe Rate an gravierenden Spätschäden, so dass diese Strahlenart heutzutage nicht mehr verwendet wird. Kohlenstoffionen gehören auch zu den Hoch-LET Strahlen und eine Kombination aus Kohlenstoffionen und Photonen (bimodale Radiotherapie) ist einer alleinigen Photonenbestrahlung überlegen ohne dass es zu einer Steigerung der Komplikationsrate kommt. Diese Kombinationstherapie ist der Therapiestandard, wenn der Patient Zugang zu einer Bestrahlung mit Kohlenstoffionen hat.

Zur alleinigen Bestrahlung mit Kohlenstoffionen sind bisher keine Daten aus Europa vorhanden. Die vorliegende ACCO-Studie (Adenoid cystic Carcinoma and Carbon ion Only irradiation) ist eine prospektive, offene, randomisierte, zweiarmige Phase II investigator-initiated Studie, die die alleinige Radiotherapie von Kohlenstoffionen in dieser Tumorentität im Vergleich zur bimodalen Radiotherapie untersucht. Im experimentellen Arm A wird die Bestrahlung innerhalb von circa 4 Wochen (22 Fraktionen) appliziert, während der Standardarm (Arm B) mit der Kombinationstherapie sich über 6 1/2 Wochen (33 Fraktionen) erstreckt. Ziel der Studie ist die Prüfung, ob die lokoregionäre Kontrolle nach alleiniger Bestrahlung mit Kohlenstoffionen bei Patienten, die keine Lymphknotenmetastasen nachgewiesen haben, diejenige einer Kombinationstherapie weiter verbessert. Das Follow-up der Patienten findet bis 5 Jahre nach Therapiebeginn statt. Es sollen 314 Patienten werden. Nebenzielkriterien sind das progressionsfreies Überleben, das Gesamtüberleben, Akut- und Spättoxizität, sowie Lebensqualität. Die Studie wird von der Deutschen Krebshilfe gefördert und begann die Rekrutierung im Dezember 2019.

3.0 Hintergrund

Adenoidzystische Karzinome sind seltene Tumore (Inzidenz 0,14 – 0,64 / 100.000), die meistens in der Kopf-Hals Region (große und kleine Speicheldrüsen, Nasenschleimhaut) auftreten und etwa 10-15% der malignen Speicheldrüsentumore ausmachen. Sie haben ein langsames Wachstum neigen aber aufgrund ihres Wachstumsmusters zu lokalen Rezidiven, weshalb insbesondere bei Vorhandensein von Risikofaktoren (pT3/4, R1/2, Pn1) eine Nachbestrahlung indiziert ist. Eine lymphogene Metastasierung wird in 19% der Speicheldrüsen ACCs gefunden (GROUP 2017). Die lymphogene Metastasierungsrate bei ACCs des Nasopharynx, der Nasennebenhöhlen, der Tränendrüse oder der äußeren Gehörgangs liegt bei lediglich 5% (GROUP 2016). Die häufigste hämatogene Metastasierung erfolgt in die Lunge mit einer Inzidenz von 35 – 50% (Bradley 2004). Weitere Metastasierungsorte sind Leber und Knochen. Während Patienten mit Lungenmetastasen relativ lange asymptomatisch weiterleben können und die lokale Kontrolle des Primärherds entscheidend für die Lebensqualität ist, sinkt das Gesamtüberleben bei Knochen und Lebermetastasierung signifikant ab (median 54 Monate auf median 20 Monate; (Sung, Kim et al. 2003)).

3.1 Radiotherapie mit Photonen

Aufgrund der Seltenheit der Tumore handelt es sich meist um retrospektive Analysen einzelner Kliniken oder Institute, die sich über einen sehr langen Zeitraum mit sich ändernder Bestrahlungstechnik erstrecken. Eigene Erfahrungen unter Verwendung moderner Photonentechniken mit einer medianen Dosis von 66 Gy (Ed 1,8 – 2 Gy) ergab bei 37 Patienten eine lokale Kontrollrate nach 3 Jahren von 56% und nach 5 Jahren von 40% (Jensen, Nikoghosyan et al. 2015). 30% der Patienten hatten eine akute Grad 3 Toxizität der Schleimhäute. Höher-gradige Spätreaktionen waren lediglich in 3% (Hörverschlechterung) zu berichten (Jensen, Nikoghosyan et al. 2015). Mendenhall und Kollegen berichteten von lokalen Kontrollraten nach 5 Jahren von 77%. Bei primär bestrahlten (nicht operierten) Patienten sank die Kontrollrate aber auf 56% (Mendenhall, Morris et al. 2004).

3.2 Radiotherapie mit Neutronen

Neutronen gehören zu den Ionenstrahlen mit einem hohen linearen Energietransfer (Hoch-LET). Adenoidzystische Karzinome galten lange Zeit als die „Paradeindikation" für eine Neutronenbestrahlung, da hiermit die besten Kontrollraten erzielt werden konnten. Bei einer südafrikanischen Serie mit 108 Patienten mit ACC und makroskopischem (Rest-)Tumor lag die 5-Jahres lokale Kontrolle bei 52% (Stannard, Vernimmen et al. 2013). Am dkfz wurden 29 Patienten mit makroskopischem ACC mit Neutronen behandelt. Die 5-Jahres lokale Kontrolle lag bei 75%. Das Hauptproblem der Neutronenbestrahlung sind die Spätkomplikationen. Bei der Analyse von 335 mit Neutronen behandelten Speicheldrüsentumoren lagen das aktuarische Risiko einer Grad 3 oder Grad 4 Komplikation bei 20% (Stannard, Vernimmen et al. 2013). Auch bei den dkfz Patienten lag die Rate an später Grad 3/4 Toxizitäten bei 17%.

3.3 Radiotherapie mit kombinierter Modalität (IMRT + C12)

Die eigene Klinik sammelte Daten zur kombinierten Bestrahlung mit einer IMRT Photonentechnik inkl. der ersten Lymphabflussstationen und einem Kohlenstoffionenboost von 6-8 Fraktionen mit 3 Gy(RBE) Einzeldosen seit 1997. Erste Daten zu dieser Kombination wurden 2005 von Schulz-Ertner et. al. publiziert und zeigten einen Vorteil im Vergleich zur historischen alleinigen IMRT Technik der Abteilung (Schulz-Ertner, Nikoghosyan et al. 2005). 2015 wurden diese Daten von Jensen et al. aktualisiert und mit allen Patienten erweitert, die bis 2007 ihre Boost-Bestrahlung an der Gesellschaft für Schwerionenforschung (GSI) in Darmstadt erhalten hatten (Jensen, Nikoghosyan et al. 2015): Bei einer medianen Nachbeobachtungszeit von 74 Monaten zeigten die 58 Patienten (alle mit makroskopischem (Rest-)Tumor) mit einer kombinierten Therapie eine lokale Progressionsfreiheit von 84% nach 3 Jahren und 60% nach 5 Jahren und war somit signifikant besser als der historische Vergleich mit 37 Patienten die mit einer alleinigen IMRT behandelt wurden (medianes follow-up 63 Monate; lokales PFS 3 Jahre 59%, 5 Jahre 40%). Dieser Vorteil spiegelte sich dann auch in einem signifikanten Überlebensvorteil wider: Medianes Gesamtüberleben in der Kombinationsbehandlung 102 Monate gegenüber 74 Monate nach alleiniger IMRT (p= 0,015) (Jensen, Nikoghosyan et al. 2015). Die COSMIC Studie untersuchte diese Methodik dann in einer prospektiven Studie, in die 53 Patienten mit malignen Speicheldrüsentumoren eingeschlossen wurden (Jensen, Nikoghosyan et al. 2010). Eine ACC Histologie war in 47 Patienten vorhanden (89%) und 33 Patienten (62%) hatten makroskopischen (Rest-)Tumor. Mit einer medianen Nachbeobachtungszeit von 42 Monaten lag die lokale Progressionsfreiheit der ACC Patienten nach 3 Jahren bei 82% und bestätigte somit die retrospektiv erhobenen Daten des zuvor erwähnten GSI Kollektivs (Jensen, Nikoghosyan et al. 2015).

Die Toxizität dieser Kombinationsbehandlung stellte keinen limitierenden Faktor dar: In der COSMIC Studie waren an schweren Akuttoxizitäten (>2°) lediglich eine Mucositis Grad 3 in 25% und eine Dermatitis Grad 3 in 10% der Patienten zu verzeichnen (Jensen, Nikoghosyan et al. 2015). Höher gradige Spät-Komplikation war eine Carotis-Blutung in einem Patient (Grad 4). Im GSI Kollektiv traten als Spätkomplikation u.a. in 10% der Patienten ein einseitiger Hörverlust, in 10% ein Geruchsverlust und bei einem Patienten (2%) eine einseitige Erblindung durch Netzhautablösung auf (Jensen, Nikoghosyan et al. 2015).
Aufgrund der erreichten Ergebnisse des GSI Kollektivs und der COSMIC Studie kann die bimodale Therapie aktuell als Standardtherapie angesehen werden.

3.4 Kohlenstoffionentherapie

Daten zur alleinigen Kohlenstoffionenbehandlung des ACC stammen aus Japan. Alle Behandlungen wurden hier mit passiver beam Modulation durchgeführt. Zur Berechnung der biologischen Dosis wurde unter Annahme von fixen spreadout Bragg peak (SOBP) Dosisprofilen durchgeführt. Dieses System unterscheidet sich vom local effect model (LEM) der GSI für C12 Bestrahlung mit aktivem scanning. Zur Vergleichbarkeit der Dosisangaben der Einzeldosen dienen Konversionstabellen (Steinstrater, Grun et al. 2012). Zusätzlich erfolgte in Japan die Bestrahlung mit 3-4 Fraktionen pro Woche.
Mizoe et al. publizierten Daten einer Dosiseskalation bei einer Gruppe von Speicheldrüsentumoren, von denen 68% eine ACC Histologie hatten (Mizoe, Tsujii et al. 2004). Die Dosis wurde von 18x 2,7 Gy(RBE_{HIMAC}) auf 18 x 3,9 Gy(RBE_{HIMAC}) mit je 3 Bestrahlungen pro Woche gesteigert. Bei einer späteren Gruppe erfolgten 16 Fraktionen (4x/Woche) mit 3,3-4,0 Gy(RBE_{HIMAC}). Die lokalen Kontrollraten dieser beiden Gruppen unterschieden sich nicht wesentlich und erreichten 75% nach 3 Jahren, 65% nach 4 Jahren und 60% nach 5 Jahren. Akuttoxizität > Grad 2 an den Schleimhäuten wurde nur in einem Patienten beschrieben (3%). Kein Patient entwickelte eine Spätreaktion > 2°.
In einer späteren Serie mit 113 ACC Patienten wurden 16 Fraktionen mit 3,7 – 4,0 Gy (RBE_{HIMAC}) appliziert (Ikawa, Koto et al.). Hierbei wurden 3-, 4-, 5-Jahres lokale Kontrollraten von 89%, 82% und 69% erzielt. 37 Patienten (33%) hatten akute Grad 3 Toxizitäten an den Schleimhäuten. Spätreaktionen ≥ Grad 3 wurden bei 27 Patienten (24%) beschrieben (Grad 3: Katarakt (10), Osteoradionekrose (7), Hirnnekrose (2), Glaukom (2), Sehverschlechterung (2), Schleimhautgeschwür (2); Grad 4: Hirnnekrose (3), Erblindung (5)).

Tabelle: Japanische Studien zur alleinigen C12 Bestrahlung eines ACC:

Autor	n	ED [Gy(RBE_{HIMAC})]	Fx	GD [Gy(RBE_{HIMAC})]	Entspricht ED [Gy (RBE_{LEM})]	BED 2 Gy (α/β=3,5)	5J LC [%]	Spät Tox ≥3
Ikawa (Ikawa, Koto et al.)	113	3,7-4,0	16	57,6 - 64,0	4,2 – 4,4	92 - 101	69%	24%
Mizoe (Mizoe, Tsujii et al. 2004)	17	2,7 – 3,9	18	48,6 – 70,2	3,0 – 3,9	64 - 94	75%	0%
Mizoe (Mizoe, Tsujii et	19	3,3 – 4,0	16	52,8 – 64,0	3,7 – 4,4	87- 101	50%	0%

al. 2004)								

Die Universitätsklinik Heidelberg hat Erfahrungen mit einer alleinigen Bestrahlung mit C12 Ionen primär in der Rezidivsituation nach vorangegangener Bestrahlung gesammelt (Jensen, Poulakis et al. 2015): Bei 52 Patienten wurden mit einer medianen Vorbelastung von 66 Gy (20 -115 Gy) erneut unter Verwendung von Kohlenstoffionen bestrahlt. Die mediane Dosis belief sich auf 51 Gy(RBE) in 17 Fraktionen. In der Nachbeobachtung von median 14 Monaten traten zerebrale Radionekrosen 3. Grades in 2 Patienten (4%), eine drittgradige Dysphagie in einem Patienten (2%) und 2 Patienten erlitten Grad 4 Carotisblutungen. Die Rate an schwerwiegenden Spätkomplikationen lag bei 6,5%. Insbesondere die Carotisblutungen sind am ehesten auf die sehr hohe kumulative Dosis von Vorbelastung und aktueller Bestrahlung (150 Gy und 180 Gy) zurückzuführen. Die lokale Tumorkontrolle war entsprechend der niedrigeren Bestrahlungsdosis in der Re-Bestrahlungssituation bei lediglich 70% nach 1 Jahr (Jensen, Poulakis et al. 2015).

4.0 Studienziele

4.1 Primäres Studienziel

- Lokoregionäre Tumorkontrolle gemäß MRT Bildgebung nach 5 Jahren

4.2 Sekundäre Studienziele

- Progressionsfreies Überleben nach 3 und 5 Jahren
- Gesamtüberleben nach 3 und 5 Jahren
- Akuttoxizität gemäß NCI CTC AE (Version 5.0) während und bis 6 Wochen nach Radiotherapie (Rate an Toxizität > 2°)
- Spättoxizität gemäß NCI CTC AE (Version 5.0) ab 6 Wochen nach Radiotherapie (Rate an Toxizität > 2°)
- Lebensqualität gemäß QLQ-C30 und QLQ-H&N35 Fragebögen

5.0 Studiendesign

Prospektive, offene, randomisierte zweiarmige Phase II Studie

6.0 Teilnehmende Prüfärzte/Prüfzentren

Die Studie soll als monozentrische Studie durchgeführt werden. Das Zentrum für diese Studie ist:

Klinik für Radioonkologie und Strahlentherapie
Ärztlicher Direktor: Prof. Dr. Dr. Jürgen Debus
Universität Heidelberg
Im Neuenheimer Feld 400, 69120 Heidelberg
Tel.: +49-6221-568201
Fax: +49-6221-565353

Das Zentrum hat mit dem Heidelberger Ionenstrahl-Therapiezentrum (HIT) und dem Marburger Ionenstrahl-Therapiezentrum (MIT) zwei Standorte. Patienten sollen an beiden Standorten rekrutiert und behandelt werden.
Studienleitung für die Strahlentherapie ist Herr Prof. Dr. Klaus Herfarth, Klinik für Radioonkologie und Strahlentherapie der Universität Heidelberg.
Die Studienkoordination erfolgt durch Herrn Dr. Sebastian Adeberg, Klinik für Radioonkologie und Strahlentherapie der Universität Heidelberg.
Zusätzliche Studienärzte sind die mit der Behandlung am Heidelberger Ionenstrahl-Therapiezentrum (HIT) und Marburger Ionenstrahl-Therapiezentrum (MIT) betrauten Ärzte der Klinik für Radioonkologie und Strahlentherapie der Universität Heidelberg.
Die biometrische Planung erfolgt durch Prof. Dr. Meinhard Kieser, Institut für Medizinische Biometrie und Informatik der Universität Heidelberg.

7.0 Auswahl der Patienten

7.1 Einschlusskriterien

- Histologisch gesichertes adenoidzystisches Karzinom im Kopf-Hals Bereich
- Indikation zur Bestrahlung:
- nicht-operabel *und/oder*
- R1/R2 reseziert *und/oder*
- Perineuralscheiden Invasion (Pn+) *und/oder*
- pT3/pT4
- Patientenaufklärung und schriftliche Einwilligung
- KI > 60% oder ECOG 0/1 (mindestens: Selbstversorgung. Normale Aktivität oder Arbeit nicht möglich)
- Alter 18-80 Jahre
- Ausreichend wirksame Empfängnisverhütung

7.2 Ausschlusskriterien

- Ablehnung der Studie durch den Patienten
- Patient ist nicht einwilligungsfähig
- Stadium IV (Fernmetastasen) mit Ausnahme von pulmonalen Metastasen ≤ 1 cm
- Lymphknotenbefall (klinisch oder pathologisch)
- Frühere Strahlentherapie im Kopf-Hals Bereich
- Aktive Medizin-Implantate, für die zum Zeitpunkt der Behandlung keine Genehmigung für eine Ionenbestrahlung existiert (z.B. Herzschrittmacher, Defibrillator,...)
- Kontraindikation gegenüber MRT Bildgebung
- Gleichzeitige Teilnahme an einer anderen klinischen Studie, die die Ergebnisse dieser Studie oder der anderen Studie beeinflussen könnte
- Schwangerschaft

8.0 Patientenaufnahme/Registrierung

In der Studienzentrale wird ein Logbuch geführt, in dem alle Patienten, die die Auswahlkriterien erfüllen, konsekutiv erfasst und auf einem Erfassungsbogen dokumentiert werden. Falls keine Aufnahme in die Studie erfolgt, wird der Grund hier dokumentiert. Alle Patienten, die die Auswahlkriterien erfüllen, die aufgeklärt wurden und ihr Einverständnis zur Teilnahme an der Studie gegeben haben, werden in der Studienzentrale als rekrutiert registriert und randomisiert. Hierfür werden der Erfassungsbogen und die unterschriebene Einverständniserklärung an die Studienzentrale übergeben oder gefaxt. In der Studienzentrale wird der Erfassungsbogen auf Vollständigkeit überprüft und der Patient wird im Logbuch aufgenommen.

Die Aufklärung jedes Patienten erfolgt durch ein Gespräch zwischen dem Studienarzt und dem Patienten vor Einschluss in die Studie. Der Studienarzt hat dem Patienten ausreichend Bedenkzeit und Gelegenheit für Rückfragen einzuräumen und muss sich davon überzeugen, dass die Aufklärung vom Patienten verstanden wurde. Alle Fragen des Patienten müssen beantwortet und eventuelle Unklarheiten beseitigt werden. Die Einwilligung des Patienten muss sich ausdrücklich auch auf die Erhebung und Verarbeitung von personenbezogenen Daten beziehen. Deshalb werden die Patienten explizit über Zweck und Umfang der Erhebung und die Verwendung dieser Daten, insbesondere von Gesundheitsdaten informiert. Es wird schriftlich auf die Speicherung von vollständigen Namen, Geburtsdaten, Adressen, Telefonnummern in der Studienzentrale hingewiesen.

Der Patient kann jederzeit und ohne Angabe von Gründen die Einwilligung zurückziehen und die Studie abbrechen. Er wird in einem solchen Fall gebeten, den Abbruchgrund zu nennen, jedoch darauf hingewiesen, dass er dies nicht tun muss.

9.0 Behandlungsplan

- Eignungsprüfung des Patienten, Indikationsstellung
- Aufklärung des Patienten
- Randomisierung
- Erfassung der Anamnese und des körperlichen Befundes (Karnofsky-Index, Größe, Gewicht)
- Erfassung der Lebensqualität (QLQ-C30 und QLQ-H&N35)
- Aktuelle MRT Bildgebung inkl. T1/T2 Fat-Sat Aufnahmen (nicht älter als 2 Monate)
- CT Thorax (nicht älter als 3 Monate)
- CT/Sonographie Leber (nicht älter als 3 Monate)
- Knochenszintigraphie (optional)
- Zahnsanierung und Anfertigung einer Fluoridierungsschiene bei Bestrahlungen im Kieferbereich

9.1 Strahlentherapie

Die Strahlentherapie wird am Heidelberger Ionenstrahl-Therapiezentrum und am Marburger Ionenstrahl-Therapiezentrum unter alleiniger Verwendung von Kohlenstoffionen (experimenteller Arm A) bzw. einer bimodalen Radiotherapie (Kontrollarm, Arm B) durchgeführt. Im experimentellen Arm werden insgesamt 22 Fraktionen in ca. 4 Wochen appliziert (5-6 Fraktionen pro Woche) Dies entspricht einer deutlichen Verkürzung der Behandlungszeit im Kontrollarm (33 Fraktionen in ca. 6 Wochen). Die relative biologisch effektive Dosis errechnet sich aus der physikalischen Dosis unter Verwendung von LEM1 und einem alpha/beta Wert von 2.

9.2 Bestrahlungsplanung

Der Patient wird mittels Thermoplastmaske gelagert. Die Computertomographie muss nativ in 3 mm Schichten erfolgen und wenn möglich auch mit Kontrastmittel durchgeführt werden.

9.3 Konturierung

Folgende Strukturen sollten konturiert werden:

<u>Zielstrukturen experimenteller Arm A</u>:

**GTV**	Makroskopische Tumorausdehnung gemäß MRT zum Zeitpunkt der Therapieplanung
**GTVinit**	initiales GTV: präoperative Tumorausbreitung bei operierten Patienten
**CTV_BP**	GTV & ggf. GTVinit plus 6 mm Saum (9 mm entlang der perineuralen Ausbreitungswege) unter Berücksichtigung anatomischer Grenzen
**CTV_GP**	CTV_BP plus 6 mm Saum (12 mm entlang der perineuralen Ausbreitungswege) unter Berücksichtigung anatomischer Grenzen; dabei teilweise miterfasste Lymphknotenstationen sollten komplett miteingefasst werden

Zielstrukturen Kontrollarm (Arm B):

**GTV**	Makroskopische Tumorausdehnung gemäß MRT zum Zeitpunkt der Therapieplanung
**GTVinit**	initiales GTV: präoperative Tumorausbreitung bei operierten Patienten
**CTV_BP**	GTV & ggf. GTVinit plus 6 mm Saum (9 mm entlang der perineuralen Ausbreitungswege) unter Berücksichtigung anatomischer Grenzen
**CTV_GP**	CTV_BP plus 6 mm Saum (12 mm entlang der perineuralen Ausbreitungswege) unter Berücksichtigung anatomischer Grenzen und abfließende Lymphwege (nur Level II und III) Bei Mittellinienüberschreitung beidseitig

Risikostrukturen:

- Hirnstamm
- Rückenmark
- Innenohr
- Temporalpole
- Chiasma opticum
- Sehnerven bds.
- Augenbulbus
- Linse
- Tränendrüse
- Kiefergelenke
- Gll. parotis
- Gll. submandibularis

9.4 Dosisspezifikation

Ziel der Optimierung des Bestrahlungsplanes ist es, eine möglichst homogene und konformale Dosisverteilung und eine maximale Schonung der Risikoorgane zu erreichen. Die maximale Belastung der Risikoorgane darf die TD 5/5 (toxische Dosis, die 5% schwere Komplikationen in 5 Jahren verursacht) der jeweiligen Organe nicht überschreiten. Auf die Schonung von Rückenmark, Chiasma und Hirnstamm wird eine hohe Priorität gesetzt. Die Schonung der jeweiligen Sehnerven sollte entsprechend der initialen Tumorausbreitung in Absprache mit dem Patienten erfolgen

Tabelle 1: Die Dosierungen beziehen sich auf 95% des Volumens. Bei Kohlenstoffionen basiert die biologische Dosis auf dem local effect model (LEM) 1 unter Berücksichtigung eines alpha/beta Wertes für alle Gewebe von 2.

	**Experimenteller Arm A Alleinige C12-Bestrahlung**		**Kontrollarm B Bimodale RT (Photonen&C12)**	
	**CTV_GP**	**CTV_BP**	**CTV_GP**	**CTV_BP**
Einzeldosis	3 Gy(RBE)	3 Gy(RBE)	2 Gy	3 Gy(RBE)
Gesamtdosis	51 Gy(RBE)	15 Gy(RBE)	50 Gy	24 Gy (RBE)
BED2Gy*	61 Gy	18 Gy	50 Gy	29 Gy

*Die Äquivalenzdosis BED2Gy ist die Dosis, die der applizierten Dosis in konventioneller Fraktionierung (d.h. mit 2 Gy Einzeldosis entspricht; Berechnung mit einem α/β = 3 Gy für ACC).

9.5 Risikoorgane

Bei der Einhaltung der Toleranzdosen ist der Fraktionierungseffekt bei unterschiedlicher Teilbelastung bei Grundplan und Boostplan entsprechend zu berücksichtigen. Hierbei gilt die Referenzdosis von 2 Gy und ein α/β = 2 Gy für die Risikoorgane.

Tabelle 2: Toleranzdosen der Risikoorgane

	**GD / Isodose experimenteller Arm A**	**BED2Gy***	**Kommentar**
Hirnstamm	54 Gy(RBE) / 82%	60 Gy	Maximum oberflächlich
Chiasma	49,5 Gy(RBE) / 75%	52,6 Gy	Maximum
Sehnerven**	49,5 Gy(RBE) / 75%	52,6 Gy	Maximum
Rückenmark	45 Gy(RBE) / 68%	45,3 Gy	Maximum
Parotis****	31 Gy(RBE) / 47%	26,4	Mittlere Dosis
Unterkieferknochen	60 Gy(RBE) / 90%	69,8 Gy	Maximum oberflächlich
Linse***	ALARA		
Bulbus***	ALARA		
Tränendrüse***	ALARA		
Innenohr***	ALARA		
Kiefergelenk***	ALARA		

*Die hier aufgeführten Werte gelten auch für die Kombinationstherapie bei Verwendung eines alpha/beta von 2 für die jeweiligen Risikoorgane.
**Höhere Dosen nach dokumentierter Rücksprache mit dem Patienten, dass er eine Erblindung auf der entsprechenden Seite für eine höhere Tumorkontrollwahrscheinlichkeit in Kauf nimmt, möglich.
*** ALARA = As low as reasonable achievable
**** wenn nicht im Zielvolumen (z.B. Gegenseite)

9.6 Kontrollen am Bestrahlungsgerät

Die korrekte Patientenpositionierung bei der C12 Bestrahlung.wird mittels täglicher orthogonalen Röntgenbilder Bildgebung kontrolliert und gegebenenfalls korrigiert. Die Bestrahlung im Kontrollarm erfolgt als IMRT mit Bildsteuerung, wobei die Häufigkeit der Bildsteuerung je nach Lagerungsungenauigkeit und benachbarter Risikostruktur individuell festgelegt werden kann.

9.7 Supportivmaßnahmen während der Strahlentherapie

Die Strahlentherapie wird unter ambulanten Bedingungen durchgeführt, es besteht jederzeit die Möglichkeit der stationären Aufnahme zur Supportivtherapie.
Bei Zielvolumina mit Dosisapplikation im Mundbereich, des Oro- oder Hypopharynx wird die supportive Therapie mit Mundspüllösung, Amphomoronal und Glandosane empfohlen.

10.0 Klinische Untersuchungen (Visiten)

10.1 Übersicht

Klinische Visitenanzahl und -inhalte entsprechen denen der Standardtherapie. Studienbedingter Mehraufwand ergibt sich durch das regelmäßige Ausfüllen der Lebensqualitätsbögen (circa. 15 Minuten pro Visite).

	EU	AU	FU Woche 6	FU Monat 6	FU Monat 12	FU Monat 18	FU Monat 24	FU Monat 36	FU Monat 48	FU Monat 60
Ein/Ausschlusskriterien erfüllt	x									
Schriftliche Einverständnis	x									
Anamnese/Klinischer Status	x[a]		x	x	x	x	x	x	x	x
Karnofsky-Index	x		x	x	x	x	x	x	x	x
Größe	x									
Gewicht	x		x	x	x	x	x	x	x	x
QLQ-C30 und QLQ-H&N35	x	x	x	x	x	x	x	x	x	x
Symptome/Toxizität CTC AE	x	x	x	x	x	x	x	x	x	x
Doku Staginguntersuchungen	x[b]									
MRT	x[c]		x[e]	x[e]	x[e]	x[e]	x[e]	x[e]	x[e]	x[e]
CT Thorax	x[d]				x		x	x	x	x
CT/Sonographie Leber	x[d]									
Knochenszintigraphie (optional)	x									
Terminierung FU		x								

EU: Eingangsuntersuchung; AU: Abschlussuntersuchung RT; FU: follow up; [a] inkl. OP-Bericht und Histologie; [b] Staginguntersuchung von Thorax, Leber, Knochen, MRT Kopf/Hals; [c] weniger als 2 Monate alt; [d] weniger als 3 Monate alt; [e] mit Erfassung des Remissionsstatus (Remission, Stable disease, Progress)

10.2 Eingangsuntersuchung

- Anamneseerhebung (inkl. OP-Bericht, OP-Histologie)
- Dokumentation von Staginguntersuchungen (Thorax, Leber, Knochen, MRT Kopf/Hals)
- Erhebung der Lebensqualität (EORTC QLQ-C30 und QLQ-H&N35)
- Erhebung der Symptome nach NCI CTC AE (Version 5.0) Kriterien

10.3 Abschlussuntersuchung

Am letzten Bestrahlungstag (+/- 1 Tag) findet das Abschlussgespräch mit dem Prüfarzt statt:

- Evaluation und Dokumentation der Toxizität
- Erfassung der Lebensqualität anhand EORTC QLQ-C30 und QLQ-H&N35
- Terminierung der weiteren Follow-Up Termine

10.4 Follow-Up

Die onkologische Nachsorge beginnt 6 Wochen nach Ende der Radiotherapie. Weitere Nachsorgetermine sind Monat 6, Monat 12, Monat 18, Monat 24, Monat 36, Monat 48 und Monat 60.

Hierbei werden folgende Parameter erhoben:

- Klinischer Status inkl. Karnofsky Index, Gewicht
- Erfassung der Toxizität gemäß CTC AE 5.0
- MRT Kopf/Hals mit Erfassung des Remissionsstatus (Remission, Stable disease, Progress)
- Erfassung der Lebensqualität anhand EORTC QLQ-C30 und QLQ-H&N35
- CT Thorax (jährlich)

11.0 Dauer der Studienteilnahme

Die Rekrutierung der Patienten erfolgt über einen Zeitraum von 7 Jahren, die Auswertung der Studienergebnisse bezüglich des primären Endpunkts der Studie (Lokoregionäre Kontrolle) erfolgt 60 Monate nach Bestrahlungsbeginn des letzten Patienten. Als Studienende wird das Ende der Nachbeobachtungszeit des letzten Patienten definiert (60 Monate nach Behandlungsbeginn).
Geplanter Beginn der Studie ist das 2. Quartal 2019.

first patient in	last patient in	last patient out
Q2 2019	Q2 2026	Q2 2031

11.1 Vorzeitiges Ausscheiden eines Patienten aus der Studie

Individuelle Abbruchkriterien während der Behandlungsphase:

- Widerruf der Zustimmung des Patienten zur Studienteilnahme
- bei therapieresistenten schweren Nebenwirkungen (CTC Grad 4 Toxizität, welche sich nicht spontan, nach Supportivtherapie oder nach einer Bestrahlungspause erholt)

Individuelle Abbruchkriterien während der Nachbeobachtungsphase:

- Widerruf der Zustimmung des Patienten zur Studienteilnahme
- bei Tumorrezidiv

Weiteres Vorgehen nach dem Ausscheiden:
Neben der strahlentherapeutischen Sorgfaltspflicht zur Erhebung von Spättoxizitäten werden mit Einverständnis des Patienten auch nach Studienende (nach Ablauf der studienbedingten Nachbeobachtungsphase) in Kooperation mit dem betreuenden Kopf-Hals Chirurgen Toxizitäten erfasst.

12.0 Erfassung der therapeutischen Effektivität

12.1 Zeitplan

Einreichung des kompletten Protokolls zur Begutachtung in der Ethikkommission und des Bundesamtes für Strahlenschutz ist für das erste Quartal 2018 geplant. Rekrutierungsstart ist für das zweite Quartal 2019 terminiert. Bei einer Rekrutierung von etwa 45 Patienten pro Jahr ist das Rekrutierungsende für das 2. Quartal 2026 anvisiert.

12.2 Definition eines Rezidives

Eine direkte Umsetzung der RECIST Kriterien auf die Response Evaluation ist aufgrund des Wachstumsverhaltens der der häufig anzutreffenden radiogenen Veränderungen im Bestrahlungsgebiet häufig nicht möglich. Die Response Evaluation erfolgt deshalb primär deskriptiv. Ein Rezidiv-Verdacht sollte in einer 2. Untersuchung 2-3 Monate später validiert werden.

13.0 Sicherheit

13.1 Ermittlung der Sicherheit

Die Akut- und Spättoxizität der Strahlentherapie wird während und nach der Therapie engmaschig an den jeweiligen Nachsorgeterminen anhand der NCI CTCAE Version 5.0 Kriterien erfasst.

Grad 1 = leicht (keine therapeutischen Maßnahmen erforderlich)
Grad 2 = mittelschwer (ambulante medikamentöse Therapie)
Grad 3 = schwer (stationäre Therapie)
Grad 4 = lebensbedrohend oder zu Behinderungen führend
Grad 5 = Nebenwirkung mit Todesfolge

Typische Toxizitäten und ihre Klassifizierung gemäß CTC AE V4 sind im Anhang des Protokolls aufgeführt.

13.2 Data Safety Monitoring Board

Ein unabhängiges Data Safety Monitoring Board (DSMB) wird die Rekrutierung, die gemeldeten unerwünschten Ereignisse und die Datenqualität mindestens einmal pro Jahr kontrollieren. Basierend auf seinen Bericht, wird das DSMB dem Studienleiter Empfehlungen bezüglich der Veränderung, Fortsetzung oder Beendigung der Studie geben. Das DSMB besteht aus unabhängigen Experten des Fachgebietes Radioonkologie (Prof. Dr. Marc Münter, Klinikum Stuttgart und Prof. Dr. Florian Sterzing, Strahlentherapie Süd, Kempten). Die Mission der DSMB wird die Gewährleistung der ethischen Durchführung der Studie, sowie die Wahrung der Sicherheitsinteressen der Patienten in der Studie sein. Probleme, welche identifiziert werden, werden mit dem PI diskutiert, welcher angemessene Maßnahmen ergreifen wird. Relevante Informationen (einschließlich relevante Sicherheitsdaten) werden in den Statusberichten der Studie aufgenommen, welche als Diskussionsgrundlage für die Studiengruppensitzungen, an welchen der PI, der Studienkoordinator und Sub-Investigators teilnehmen, dient.

14.0 Dauer der Studie/Studienabbruch

14.1 Reguläres Studienende

Reguläres Ende der Behandlungsphase: 4 Wochen nach Beginn der Strahlentherapie (alleinige C12-Bestrahlung); 6 Wochen nach Beginn der Strahlentherapie (bimodale Radiotherapie)
Reguläres Ende der Studienteilnahme: nach einer Nachbeobachtungszeit von maximal 5 Jahren

14.2 Vorzeitiges Studienende/Abbruch der gesamten Studie

Als Gründe für einen vorzeitigen Abbruch der gesamten Studie kommen in Frage:

- Entscheidung der Studienleitung bei unvertretbaren Risiken und Toxizitäten unter Nutzen-Risiko-Abwägung
- Abbrucherwägung bei jeder Grad 5 Toxizität, bei 2 konsekutiven Grad 4 Toxizitäten, bei 5 konsekutiv auftretenden Grad 3 Toxizitäten
- neue (wissenschaftliche Erkenntnisse) während der Laufzeit der Studie
- nicht adäquate Rekrutierungsrate

14.3 Individuelle Abbruchkriterien

- Widerruf der Zustimmung des Patienten zur Studienteilnahme

- Während der Behandlungsphase: bei therapieresistenten schweren Nebenwirkungen (CTC Grad 4 Toxizität, welche sich nicht spontan, nach Supportivtherapie oder nach einer Bestrahlungspause erholt)

- Während der Nachbeobachtungsphase: bei Tumorrezidiv

15.0 Biometrie

15.1 Fallzahlberechnung

Primäres Studienziel ist der Nachweis, dass bei lokoregionärer Radiotherapie mit C12 Ionen die lokale Kontrollrate nach 5 Jahren π_{C12} größer als die lokale Kontrollrate der bimodalen Radiotherapie π_{bim} ist. Kontrollraten von ca. 60% wurden bei Behandlung mit IMRT und C12 Boost beobachtet, und es wird angenommen, dass sich diese Rate unter C12 um 10% verbessern lässt (s. Kapitel 3.4). Die Nullhypothese (H_0: $\pi_{C12} \leq \pi_{bim}$; „die lokale Kontrollrate nach 5 Jahren bei lokoregionärer Radiotherapie mit C12 Ionen ist kleiner oder gleich der lokalen Kontrollrate bei bimodaler Radiotherapie") wird zum einseitigen Niveau α=0,15 getestet, für die Alternative einer lokalen Kontrollrate nach 5 Jahren bei lokoregionärer Radiotherapie mit C12 Ionen von π_{C12} =70% und bei bimodalen Radiotherapie von $\pi_{bim} = 60\%$ soll eine Power von 0,75 bestehen. Hierzu ist unter der Verwendung eines Chi²-Tests eine Fallzahl von 266 Patienten (133 pro Arm) notwendig. Unter der Annahme einer Drop-out-Rate von 15% werden 314 Patienten in die Studie eingeschlossen. Durch die Auswertung mittels eines logistischen Regressionsmodells, welches für die Faktoren „Resttumor vorhanden" und „Lungenmetastasen vorhanden" adjustiert, wird aufgrund der zusätzlich erklärten Varianz eine erhöhte Power erwartet. Dadurch, dass fehlende Werte mittels multipler Imputation ersetzt werden sollen (siehe Abschnitt 15.4), ist ebenfalls von einer zusätzlich erhöhten Power auszugehen. Die Berechnungen wurden mit nQuery Advisor, Version 7.0, durchgeführt.

15.2 Randomisierung

Nacheinander gescreente und teilnahmeberechtigte Patienten werden nach Studienstart in die Studie eingeschlossen. Um vergleichbare Behandlungsgruppen (Arm A: experimentelle Gruppe: lokoregionäre Strahlentherapie mit nur C12 Ionen; Arm B: Kontrollgruppe: bimodale Strahlentherapie) bezüglich bekannter („Resttumor vorhanden" (ja/ nein) und „Lungenmetastasen vorhanden" (ja/ nein)) und unbekannter Risikofaktoren zu erhalten, wird jeder Patient zufällig zu den Behandlungsgruppen in balancierten permutierten Blöcken und stratifiziert nach Resttumor und Lungenmetastasen mit Hilfe der web-basierten Software *randomizer.at* (durch das Institut für Medizinische Informatik, Statistik und Dokumentation der Medizinischen Universität Graz (https://www.randomizer.at) zur Verfügung gestellt) zugeteilt. Laut Fallzahlplanung sollen 314 Patienten randomisiert werden.

15.3 Studienpopulation

Intent-To-Treat-Population (ITT): Die ITT-Population umfasst alle randomisierten Patienten, welche in jenem Therapiearm ausgewertet werden, zu dem sie randomisiert worden sind. Die ITT-Population ist das primäre Auswertungskollektiv für alle Wirksamkeitsendpunkte und Patientencharakteristika.

Per-Protokoll-Population (PP): Alle Patienten der ITT-Population, welche die geplante Therapie vollständig erhalten haben und deren Dokumentation zumindest soweit vollständig ist, dass aus den Daten Schlussfolgerungen über die Wirksamkeit abgeleitet werden können. Die Analysen der PP-Populationen dienen als Sensitivitätsanalysen, anhand deren die Robustheit der Ergebnisse aus der ITT-Population untersucht werden soll.

Safety Population: Alle Patienten der ITT-Population, bei denen die geplante Therapie begonnen wurde (mind. 1 Tag). Patienten in der Safety-Population werden in jenem Therapiearm ausgewertet, gemäß dem sie behandelt worden sind. Dies ist das primäre Auswertungskollektiv für sämtliche Safety-Endpunkte.

Die Zuordnung jedes Patienten zu den verschiedenen Analysepopulationen (ITT-, PP-, Safety-Population) wird im statistischen Analyseplan definiert und ausführlich erläutert.

15.4 Auswertung

Der primärer Endpunkt „Lokoregionäre Tumorkontrolle gemäß MRT Bildgebung nach 5 Jahren" wird mittels einem logistischen Regressionsmodell ausgewertet, welches für die Faktoren („Resttumor vorhanden" (ja/nein) und „Lungenmetastasen vorhanden" (ja/nein) adjustiert. Die Nullhypothese wird hierbei zum einseitigen Niveau von α=0,15 getestet. Mit dem gegenüber einer konfirmatorischen Phase-II-Studie erhöhten Signifikanzniveau wird zum Einen dem Phase-II-Charakter der Studie Rechnung getragen und zum Anderen der in einem überschaubaren Zeitrahmen rekrutierbaren Patientenzahl, mit der zu diesem Niveau eine ausreichend hohe Power erzielt werden kann. Zusätzlich wird das zugehörige Odds Ratio für den Therapieeffekt mit einem zweiseitigen 70%-Konfidenzintervall angegeben. Fehlende Werte des primären Endpunkts werden mittels multipler Imputation ersetzt, hierbei werden unter Verwendung der „fully conditional specification"- Methode (van Buuren, 2007) die Faktoren „Therapiegruppe" (experimenteller Arm A/Kontrollarm B), „Resttumor vorhanden" (ja/nein) und „Lungenmetastasen vorhanden" (ja/nein)" im Imputationsprozess berücksichtigt. Als Sensitivitätsanalysen erfolgen eine Complete-Case-Analyse, sowie die Durchführung einer Best- und Worst-Case-Analyse. Ergänzend wird die Time-to-event-Kurve für das Hauptzielkriterium mit der Methode nach Kaplan und Meier (1958) geschätzt.

Die Studie wird von einem unabhängigen Data Safety Monitoring Board (DSMB) begleitet, das auf der Basis der akkumulierten Daten das Nutzen-Risiko-Profil der untersuchten Therapie im Studienverlauf bewertet und ggf. die Studienleitung informiert, sofern aus Sicht des DSMBs Änderungen an der Studiendurchführung als notwendig erachtet werden. Die entsprechende Einschätzung und ggf. eine daraus resultierende Empfehlung des DSMBs wird der Studienleitung übermittelt.

Die Nebenzielkriterien werden mit Methoden der deskriptiven Datenanalyse ausgewertet (Abt, 1987). Entsprechend des Skalierungsniveaus werden für ordinal- und intervallskalierte Variablen geeignete Lage- und Streuungsmaße der empirischen Verteilung angegeben, für nominalskalierte Endpunkte werden absolute und relative Häufigkeiten berechnet. Für Time-to-event-Endpunkte wird nach der Kaplan-Meier-Methode die Wahrscheinlichkeit für das Eintreten des jeweiligen Zielereignisses in Abhängigkeit von der Zeit geschätzt. Es werden darüber hinaus deskriptiv zu interpretierende zweiseitige 95%-Konfidenzintervalle – bei Time-to-event-Endpunkten unter adäquater Berücksichtigung von zensierten Beobachtungen – berechnet und angegeben. Als Safety-Analyse werden für unerwünschte Ereignisse (AEs) und schwerwiegende unerwünschte Ereignisse (SAEs) relative und absolute Häufigkeiten zusammen mit 95%-Konfidenzintervallen tabelliert. Weitere Details der Auswertung werden in einem statistischen Analyseplan spezifiziert, der vor Datenbankschluss finalisiert werden wird. Alle Berechnungen werden mit SAS Version 9.4 oder höher durchgeführt.

16.0 Datenmanagement

16.1 Patientenidentifikationsliste

Alle patientenbezogenen Daten werden in pseudonymisierter Form erfasst. Jeder Patient ist durch eine Patientenidentifikationsnummer unverwechselbar gekennzeichnet. Der Prüfarzt führt eine Patientenidentifikationsliste, in der die Patientenidentifikationsnummern mit dem vollen Patientennamen verbunden sind. Diese Liste ist absolut vertraulich zu behandeln und darf das Prüfzentrum nicht verlassen. Die Patientenidentifikationsliste ist nach Studienende mindestens 15 Jahre zu archivieren.

16.2 Datenerhebung/Dokumentationsbögen

Die Datenerhebung erfolgt anhand von Prüfbogen (Dokumentationsbögen/Case Report Forms). Die Bögen sind mit Kugelschreiber auszufüllen. Eintragungen mit Bleistift oder Tinte sind nicht erlaubt. Korrekturen sind wie folgt vorzunehmen: Der falsche Eintrag wird mit einer einfachen Linie durchgestrichen, die korrekte Information daneben eingetragen und vom Prüfarzt mit Datum paraphiert und ggf. mit Angabe des Grundes der Korrektur versehen. Datenfelder, die wegen fehlender Information nicht ausgefüllt werden können, sind zu kommentieren.
Die Bögen sind zeitnah auszufüllen und anschließend vom Prüfarzt zu kontrollieren, mit Datum zu unterschreiben und der Studienzentrale zuzuleiten.

16.3 Aufbewahrung der Studienunterlagen

Die Originale aller zentralen Studiendokumente einschließlich Dokumentationsbogen werden in der Studienzentrale für mindestens 30 Jahre nach Erstellung des Abschlussberichtes aufbewahrt.
Der Prüfer/Leiter des Prüfzentrums bewahrt die angefallenen administrativen Dokumente (Schriftverkehr mit der Ethikkomission, Studienleitung, Studienzentrale), die Patientenidentifikationsliste, die unterschriebenen Einwilligungserklärungen, Kopien der Dokumentationsbögen und der allgemeinen Studiendokumentation (Protokoll, Amendments) für die oben genannte Zeit auf.
Originaldaten der Studienpatienten (Krankenakten) sind entsprechend der für das Prüfzentrum gültigen Archivierungsfrist, aber nicht weniger als 30 Jahre, aufzubewahren.

16.4 Datenschutz

Im Rahmen der Studie werden von den Studienteilnehmern personenbezogene Daten (vollständiger Name, Geburtsdatum, Adresse) und Daten zur Behandlung und zum Krankheitsverlauf (medizinische Befunde, Behandlungsarten, verordnete Medikamente, etc.) erhoben. Diese Daten unterliegen der ärztlichen Schweigepflicht und den Bestimmungen des Bundesdatenschutzgesetzes (BDSG). Sie werden in pseudonymisierter* Form mit Hilfe einer Identifikationsnummer elektronisch gespeichert und ausgewertet. Eine Weitergabe von Patientendaten erfolgt ggf. nur in pseudonymisierter Form. Dritte erhalten keinen Einblick in Originalunterlagen. Ausgenommen hiervon sind Dritte die zu Qualitätszwecken Einsicht in die Studiendokumente sowie deren Quelldaten erhalten (vgl. 16.5).

* Pseudonymisierung ist die Verarbeitung personenbezogener Daten in einer Weise, dass die personenbezogenen Daten ohne Hinzuziehung zusätzlicher Informationen („Schlüssel“) nicht mehr einer spezifischen betroffenen Person zugeordnet werden können. Diese zusätzlichen Informationen werden dabei gesondert aufbewahrt und unterliegen technischen und organisatorischen Maßnahmen, die gewährleisten, dass die personenbezogenen Daten nicht einer identifizierten oder identifizierbaren natürlichen Person zugewiesen werden.

16.5 Internes Monitoring

Zur Sicherstellung der Datenqualität und -konsistenz werden durch das leitende Studienzentrum in Heidelberg Maßnahmen zur internen Qualitätskontrolle durchgeführt. Dazu werden regelmäßig bei 10% aller bis zu diesem Zeitpunkt eingeschlossenen Patienten (per Zufallsprinzip ausgewählt) durch einen internen Monitor im Rahmen der Qualitätssicherung (internes Monitoring) folgende Inhalte kontrolliert:

- Erfüllung der Ein- und Ausschlusskriterien,
- Behandlung entsprechend des Studienprotokolls (Behandlungsarm, medizinische Behandlung und Bestrahlungsplanung),
- Follow-ups entsprechend des Protokolls (Zeitpunkte, Diagnostik, Evaluation der Tumorgröße und Nebenwirkungen),
- Überprüfung der vorschriftsmäßigen Dokumentation und Meldung von AEs/SAEs.
- Überprüfung der Rekrutierungs- sowie Drop-out Rate.
- Überprüfung der Dokumentation in der HIRO Datenbank
- Sammlung von Material für translationalen Aspekt der Studie.

Nach jedem internen Monitoring erhält der Studienleiter einen Monitoringbericht. Dieser wird im ISF aufbewahrt.

17.0 Ethische Grundlagen

17.1 Deklaration von Helsinki

Die Untersuchung wird in Übereinstimmung mit der Deklaration von Helsinki in ihrer aktuellen Fassung durchgeführt.

17.2 Ethikkommission

Studienprotokoll, Patienteninformation und Einwilligungserklärung werden der Ethikkommission der Medizinischen Fakultät der Universität Heidelberg zur berufsrechtlichen Beratung vorgelegt. Die Studie wird erst nach Erhalt des zustimmenden Votums begonnen. Die Ethikkommission wird vom Studienleiter über alle Änderungen im Studienprotokoll, die die Sicherheit der Patienten beeinträchtigen könnten, umgehend informiert.

17.3 Aufklärung der Patienten

Vor Aufnahme in die Studie wird jeder Patient vom behandelnden Arzt über Wesen, Ziele, erwartete Vorteile und mögliche Risiken der Studie informiert. Die Teilnahme an der Studie ist freiwillig und kann jederzeit, ohne Nennung von Gründen und ohne Nachteile für die weitere medizinische Versorgung widerrufen werden.
Jeder Patient muss seine (schriftliche) Einwilligung zur Teilnahme an der Studie erklären. Dem Patienten muss dabei ausreichend Zeit und Gelegenheit gegeben werden, um vor der Einleitung der Studienmaßnahmen über seine Teilnahme zu entscheiden und offene Fragen zu klären. Die Einwilligungserklärung wird vom Patienten und vom behandelnden Arzt unterzeichnet.

17.4 Strahlentherapeutische Aufklärung

Die Patienten erhalten unabhängig von der Aufklärung zur Studienteilnahme eine gesonderte Strahlentherapieaufklärung, bei der auf die spezifischen Risiken der zu bestrahlenden Region eingegangen wird. Diese Aufklärung wird auf den hierzu gebräuchlichen Formularen dokumentiert und zusammen mit dem Bestrahlungsplan und -protokoll aufbewahrt.

17.5 Verwendung, Speicherung und Weitergabe von Daten

Die Vorschriften über die ärztliche Schweigepflicht und den Datenschutz werden gewahrt. Die Patienten werden darüber informiert, dass ihre krankheitsbezogenen Daten in pseudonymisierter Form gespeichert und für wissenschaftliche Auswertungen (Publikationen, Zulassungsdossiers) verwendet werden. Die Patienten haben das Recht, über die gespeicherten Daten informiert zu werden. Eine Einsicht der Originalkrankenakten durch Dritte geschieht nur durch die Studienleitung oder Beauftragte staatlicher Behörden (Überwachungsbehörden). Die mit der Datenüberprüfung beauftragten Personen sind zur strengen Vertraulichkeit und zur Beachtung des Datenschutzes verpflichtet.
Bei Rücktritt von der Studie wird bereits gewonnenes Datenmaterial vernichtet, wenn der Patient einer weiteren Verwendung der Daten nicht zugestimmt hat.

18.0 Gesetzliche und administrative Regelungen

18.1. GCP

Die Empfehlungen der Guten Klinischen Praxis (siehe ICH-GCP: International Conference on Harmonisation - Good Clinical Practice; 01.05.1996) werden berücksichtigt.

18.2 Gesetzliche Grundlagen

Die gesetzlichen Vorgaben der Strahlenschutzverordnung (Bundesgesetzblatt 2001 Teil I Nr. 38, vom 26.07.2001, zuletzt geändert durch Artikel 5 Absatz 7 des Gesetzes vom 24.02.2012), der Röntgenverordnung und der Richtlinie zum Strahlenschutz werden eingehalten.
Die Studienleitung kann eine mindestens zweijährige Erfahrung in klinischen Studien vorweisen.

18.3 Strahlenschutz und Versicherung

Die Studie wurde beim Bundesamt für Strahlenschutz (BfS) gemeldet. Das BfS hat am 24.09.2018 einer Therapie gemäß des experimentellen Arms zugestimmt. Das BfS wird über die im Amendement vom 24.9.2018 festgelegte Randomisierung gegen einen Kontrollarm in Kenntnis gesetzt. Zusätzlich wird eine Strahlenschutzversicherung für die Patienten abgeschlossen (Versicherer: HDI-Gerling Industrie Versicherung AG, Versicherungsnummer 57 010310 03018). Die strahlenbelastenden studienbedingten Maßnahmen werden erst begonnen, wenn die erforderlichen Genehmigungen des BfS vorliegen. Die Genehmigungspflicht basiert auf der Verwendung von Kohlenstoffionen. Alle radiologischen Untersuchungen zum Staging während der Nachbeobachtungsphase entsprechen der klinischen Routine.

18.4 Finanzierung

Die Radiologische Universitätsklinik, Abteilung RadioOnkologie und Strahlentherapie, Universitätsklinikum Heidelberg, INF 400, 69120 Heidelberg, wird die Kosten der klinischen Prüfung übernehmen. Die Studie wird zur Finanzierung bei der Deutschen Krebshilfe eingereicht. Hierbei werden Personalmittel für die behandelnden Ärzte, die Study Nurses, das Datenmanagement und das Projektmanagement beantragt. Ferner werden Druckkosten, Reisekosten (für das DSMB sowie den Austausch HIT/MIT), das Monitoring, die Gebühren der Ethikkommission und des Bundesamtes für Strahlenschutzes, als auch für die Probandenversicherung beantragt.
Die Studienteilnahme wird für die Patienten keine zusätzlichen Kosten (im Vergleich zur Standardbehandlung) verursachen. Alle beteiligten Personen (u.a. Studienleiter, -koordinator) erklären, dass kein Interessenskonflikt im Zusammenhang mit der Durchführung und Auswertung der Studie besteht.

18.5 Publikation

Es ist vorgesehen, dass die Ergebnisse der Studie publiziert werden. Die Reihenfolge der Autorenschaft wird von der Studienleitung festgelegt.

18.6 Einhaltung des Protokolls und Protokolländerungen

Das Studienprotokoll ist genau einzuhalten. Jede vom Prüfer zu vertretende Abweichung von den vorgesehenen Untersuchungs- und Behandlungsmaßnahmen oder -zeitpunkten ist zu dokumentieren und zu begründen (z.B. Notfallmaßnahmen). Änderungen oder Ergänzungen des Studienprotokolls können nur vom Studienleiter veranlasst und autorisiert werden.

19.0 Literatur

Abt, K. (1987). Descriptive data analysis: a concept between confirmatory and exploratory data analysis. Methods of Information in Medicine, 26: 77–88.

Bradley, P. J. (2004). "Adenoid cystic carcinoma of the head and neck: a review." Curr Opin Otolaryngol Head Neck Surg **12**(2): 127-132.

INTERNATIONAL HEAD AND NECK SCIENTIFIC GROUP (2016). "Cervical lymph node metastasis in adenoid cystic carcinoma of the sinonasal tract, nasopharynx, lacrimal glands and external auditory canal: a collective international review." J Laryngol Otol **130**(12): 1093-1097.

INTERNATIONAL HEAD AND NECK SCIENTIFIC GROUP (2017). "Cervical lymph node metastasis in adenoid cystic carcinoma of the major salivary glands." J Laryngol Otol **131**(2): 96-105.

Ikawa, H., M. Koto, R. Takagi, D. K. Ebner, A. Hasegawa, K. Naganawa, T. Takenouchi, T. Nagao, T. Nomura, T. Shibahara, H. Tsuji and T. Kamada "Prognostic factors of adenoid cystic carcinoma of the head and neck in carbon-ion radiotherapy: The impact of histological subtypes." Radiotherapy and Oncology **123**(3): 387-393.

Jensen, A. D., A. Nikoghosyan, C. Windemuth-Kieselbach, J. Debus and M. W. Munter (2010). "Combined treatment of malignant salivary gland tumours with intensity-modulated radiation therapy (IMRT) and carbon ions: COSMIC." BMC Cancer **10**: 546.

Jensen, A. D., A. V. Nikoghosyan, K. Lossner, T. Haberer, O. Jakel, M. W. Munter and J. Debus (2015). "COSMIC: A Regimen of Intensity Modulated Radiation Therapy Plus Dose-Escalated, Raster-Scanned Carbon Ion Boost for Malignant Salivary Gland Tumors: Results of the Prospective Phase 2 Trial." Int J Radiat Oncol Biol Phys **93**(1): 37-46.

Jensen, A. D., A. V. Nikoghosyan, M. Poulakis, A. Hoss, T. Haberer, O. Jakel, M. W. Munter, D. Schulz-Ertner, P. E. Huber and J. Debus (2015). "Combined intensity-modulated radiotherapy plus raster-scanned carbon ion boost for advanced adenoid cystic carcinoma of the head and neck results in superior locoregional control and overall survival." Cancer **121**(17): 3001-3009.

Jensen, A. D., M. Poulakis, A. V. Nikoghosyan, N. Chaudhri, M. Uhl, M. W. Munter, K. K. Herfarth and J. Debus (2015). "Re-irradiation of adenoid cystic carcinoma: analysis and evaluation of outcome in 52 consecutive patients treated with raster-scanned carbon ion therapy." Radiother Oncol **114**(2): 182-188.

Kaplan E.L., Meier P (1958). Nonparametric estimation from incomplete observations. JASA, 53: 457–481.

Mendenhall, W. M., C. G. Morris, R. J. Amdur, J. W. Werning, R. W. Hinerman and D. B. Villaret (2004). "Radiotherapy alone or combined with surgery for adenoid cystic carcinoma of the head and neck." Head Neck **26**: 154 – 162.

Mizoe, J. E., H. Tsujii, T. Kamada, Y. Matsuoka, H. Tsuji, Y. Osaka, A. Hasegawa, N. Yamamoto, S. Ebihara and A. Konno (2004). "Dose escalation study of carbon ion radiotherapy for locally advanced head-and-neck cancer." Int J Radiat Oncol Biol Phys **60**(2): 358-364.

Schulz-Ertner, D., A. Nikoghosyan, B. Didinger, M. Munter, O. Jakel, C. P. Karger and J. Debus (2005). "Therapy strategies for locally advanced adenoid cystic carcinomas using modern radiation therapy techniques." Cancer **104**(2): 338-344.

Stannard, C., F. Vernimmen, H. Carrara, D. Jones, S. Fredericks, J. Hille and E. de Kock (2013). "Malignant salivary gland tumours: Can fast neutron therapy results point the way to carbon ion therapy?" Radiotherapy and Oncology **109**(2): 262-268.

Steinstrater, O., R. Grun, U. Scholz, T. Friedrich, M. Durante and M. Scholz (2012). "Mapping of RBE-weighted doses between HIMAC- and LEM-Based treatment planning systems for carbon ion therapy." Int J Radiat Oncol Biol Phys **84**(3): 854-860.

Sung, M. W., K. H. Kim, J. W. Kim, Y. G. Min, W. J. Seong, J. L. Roh, S. J. Lee, T. K. Kwon and S. W. Park (2003). "Clinicopathologic predictors and impact of distant metastasis from adenoid cystic carcinoma of the head and neck." Arch Otolaryngol Head Neck Surg **129**(11): 1193-1197

Van Buuren, S. (2007). “Multiple imputation of discrete and continuous data by fully conditional specification.” Statistical methods in medical research **16**(3): 219-242.

21.0 Unterschriftenseite

____

Prof. Dr. Klaus Herfarth

Studienleitung

____

Dr. Sebastian Adeberg

Studienkoordination/Prüfarzt

____

Prof. Dr. Meinhard Kieser

Biometrie

20.0 Anhang

- Patienteninformation und Einwilligungserklärung, deutsch und englisch
- EORTC QLQ-C30 und EORTC QLQ-H&N35 Lebensqualitätsfragebogen, deutsch und englisch
- Häufigste CTC AE
